# Hemoglobin Levels as a Marker of Arrhythmia Recurrence After Atrial Fibrillation Ablation

**DOI:** 10.1002/joa3.70473

**Published:** 2026-09-21

**Authors:** Yasuharu Matsunaga‐Lee, Yasuyuki Egami, Ayako Sugino, Noriyuki Kobayashi, Masaru Abe, Hiroaki Nohara, Shodai Kawanami, Koji Yasumoto, Naotaka Okamoto, Masamichi Yano, Masami Nishino

**Affiliations:** ^1^ Division of Cardiology Osaka Rosai Hospital Osaka Japan

**Keywords:** arrhythmia recurrence, atrial fibrillation, atrial substrate, catheter ablation, hemoglobin

## Abstract

**Background:**

Low hemoglobin (Hb) is associated with hemodynamic stress and adverse cardiac remodeling. However, its clinical significance for arrhythmia recurrence after atrial fibrillation (AF) ablation remains unclear.

**Methods:**

We retrospectively studied 2202 patients undergoing AF ablation between 2014 and 2024 (radiofrequency ablation [RFA]: *n* = 1606; cryoballoon ablation [CBA]: *n* = 596). In the RFA cohort, patients were stratified by Hb quartiles, and the lowest quartile cut‐off (Hb < 12.4 g/dL) was applied to the CBA cohort. The primary endpoint was AF or atrial tachycardia recurrence beyond a 90‐day blanking period.

**Results:**

In the RFA cohort, low Hb was associated with a higher risk of arrhythmia recurrence after the index procedure (adjusted HR 1.33, 95% CI 1.07–1.65) and after multiple procedures (adjusted HR 1.57, 95% CI 1.22–2.02). No significant association was observed in the CBA cohort (adjusted HR 1.03, 95% CI 0.68–1.54). The interaction between Hb level and ablation modality was not statistically significant (*p* for interaction = 0.177). In the pooled analysis, low Hb was independently associated with arrhythmia recurrence in the total population (adjusted HR 1.24, 95% CI 1.03–1.50) and in patients with non‐paroxysmal AF (adjusted HR 1.43, 95% CI 1.11–1.85), but not in those with paroxysmal AF (adjusted HR 1.06, 95% CI 0.80–1.39).

**Conclusions:**

Lower Hb levels were associated with an increased risk of arrhythmia recurrence. The association differed according to AF type, suggesting that low Hb may reflect an adverse atrial substrate rather than a procedural effect.

## Graphical abstract

Lower hemoglobin levels are associated with increased arrhythmia recurrence after radiofrequency ablation but not cryoballoon ablation. The stronger association in patients with non‐paroxysmal AF suggests that low hemoglobin may reflect an adverse atrial substrate rather than a procedural effect.

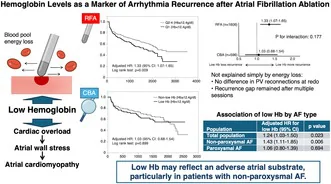

## 1 Introduction

Atrial fibrillation (AF) is the most common sustained arrhythmia, and catheter ablation has become an established rhythm control therapy [1]. Despite continuous improvements in ablation technologies, arrhythmia recurrence after pulmonary vein isolation (PVI) remains a major clinical issue. Various risk factors for recurrence have been identified, including female sex [2], long duration of AF persistence [3, 4], and enlarged left atrial size [4]. In patients with heart failure, low hemoglobin (Hb) is a well‐established marker of hemodynamic stress and poor prognosis [5]. The presence of anemia increases the cardiac overload, elevates cardiac filling pressures and enlarges atrial volume [6], all of which have been implicated in atrial cardiomyopathy [7]. These findings support a hypothesis that a mechanistic framework in which anemia reflects increased atrial wall stress, stretch‐induced structural remodeling, and impaired atrial mechanics, all of which are potent determinants of AF maintenance and ablation outcomes.

On the other hand, radiofrequency (RFA) relies on resistive heating of cardiac tissue, and the amount of delivered tissue power is influenced by impedance at the electrode–tissue and electrode–blood interfaces [8, 9]. Experimental studies have demonstrated that lower blood impedance can result in greater shunting of radiofrequency current into the blood pool, thereby reducing effective tissue heating and limiting lesion size. These observations suggest that lower Hb levels, which alter blood conductivity, may impair lesion formation during RFA and predispose to pulmonary vein (PV) reconnection and arrhythmia recurrence. In contrast, cryoballoon ablation (CBA) delivers cryothermal energy that is not dependent on electrical impedance like RFA. Lesion formation with cryoablation is less affected by blood conductivity and is primarily determined by balloon–tissue contact and cooling efficiency, as reflected by parameters such as time‐to‐isolation, nadir balloon temperature, and thawing duration, rather than by blood conductivity [10]. Therefore, if lower Hb levels are associated with recurrence after RFA but not after CBA, this would support the hypothesis that reduced lesion formation due to altered blood impedance underlies the association between Hb levels and ablation outcome.

The present study aimed to evaluate the impact of Hb levels on arrhythmia recurrence in patients undergoing AF ablation.

## 2 Methods

### 2.1 Study Population

This was a single‐center retrospective observational study. Consecutive patients who underwent their first catheter ablation of AF between September 2014 and April 2024 were included. The RFA cohort was defined as the primary analysis set, while the CBA cohort was analyzed as a secondary exploratory cohort, given the larger sample size and data availability in the RFA group. Patients without follow‐up beyond the 90‐day blanking period were excluded. The study was approved by the local institutional ethics committee and conducted in accordance with the Declaration of Helsinki (reference no. 2025‐70). Written informed consent for the ablation procedure was obtained from all participants.

Baseline Hb levels were obtained at admission prior to ablation. In the RFA cohort, patients were categorized into quartiles according to Hb values. These cut‐off values derived from the RFA population were subsequently applied to the CBA cohort for parallel analyses. Hb was also analyzed as a continuous variable.

### 2.2 Ablation Procedures

All procedures were performed under conscious sedation with continuous infusion of propofol and dexmedetomidine. After transseptal puncture, intravenous heparin was administered (initial bolus of 100 IU/kg) and the activated clotting time was maintained > 300 s throughout the procedure. The endpoint of PVI was defined as bidirectional conduction block between the left atrium and the PVs. Details of the PVI procedure using RFA or CBA were previously reported [11, 12]. The choice between RFA and CBA was left to the discretion of the treating physician.

### 2.3 Radiofrequency Ablation

PVI was performed using a three‐dimensional electroanatomical mapping system (CARTO system, Biosense Webster, Irvine, CA, USA; EnSite system, Abbott, St. Paul, MN, USA; or Rhythmia system, Boston Scientific, Marlborough, MA, USA). Additional ablation including superior vena cava isolation, ablation of non‐PV triggers, and linear ablation was performed as appropriate at the discretion of the operator. RFA energy delivery evolved over the study period in parallel with advances in catheter technology. Energy settings were as follows: non–contact force catheters, 25–30 W; contact force catheters, 25–40 W. When available, TactiFlex SE irrigated contact force catheter (Abbott, St. Paul, MN, USA) was used with 40–50 W, and ThermoCool Qdot Micro catheter (Biosense Webster, Irvine, CA, USA) was used with 50–90 W in a high‐power short‐duration setting [11]. Esophageal temperature monitoring was performed throughout the procedures, with energy delivery terminated in the event of temperature rise. Importantly, no adjustments of ablation power, contact force, or duration were made based on Hb levels during the study period.

### 2.4 Cryoballoon Ablation

CBA was performed using a 28‐mm balloon catheter (Arctic Front Advance, Medtronic, Minneapolis, MN, USA) using Ensite system (Abbott, St. Paul, MN, USA). PV occlusion was assessed by contrast injection under fluoroscopy. Complete occlusion was defined as the absence of contrast leakage into the left atrium. Phrenic nerve pacing was monitored to avoid phrenic nerve injury during right‐side PVI.

### 2.5 Follow‐Up and Outcomes

Continuous electrocardiogram (ECG) monitoring was performed during hospitalization (2–3 days). After discharge, patients underwent scheduled visits at 1, 3, 6, and 12 months, and annually thereafter. Scheduled 24‐h Holter monitoring was performed at 6 and 12 months and every 12 months thereafter. In cases of suspected recurrence, portable ECG devices were additionally used, and 7‐day Holter monitoring was actively used rather than 24‐h Holter monitoring after their availability. The primary endpoint was recurrence of AF or atrial tachycardia lasting ≥ 30 s after a 90‐day blanking period. In patients undergoing repeat ablation after RFA, PV reconnection and the number of reconnected veins were assessed.

### 2.6 Statistical Analysis

Continuous variables are presented as median (interquartile range), and categorical variables as number (percentage). Continuous variables were compared with the Mann–Whitney *U* test, and categorical variables with the *χ* ^2^ test or Fisher's exact test. Cox proportional hazards models were used to estimate hazard ratios (HRs) for recurrence according to Hb quartiles and as a continuous variable, adjusted for age, sex, AF type, chronic kidney disease, brain natriuretic peptide (BNP) and left atrial diameter. HRs for continuous variables are presented per 1‐unit increase. Kaplan–Meier curves were constructed and compared with the log‐rank test. To assess whether the association between Hb and recurrence differed by ablation modality, we constructed a pooled Cox proportional hazards model including both RFA and CBA cohorts. The model incorporated Hb group, ablation modality, and their interaction term, with adjustment for age, sex, AF type, chronic kidney disease, BNP and left atrial diameter. A two‐sided *p* < 0.10 was considered to indicate possible effect modification, whereas all other analyses used a two‐sided *p* < 0.05. All statistical analyses were performed using JMP Pro 18 (SAS Institute, Cary, NC, USA) and R version 4.5.0 (R Foundation for Statistical Computing, Vienna, Austria). Restricted cubic spline curves were generated with the survival and splines packages in R.

## 3 Results

### 3.1 RFA Cohort

A total of 1606 patients were included in the RFA cohort. The median age was 72 years (IQR 65–77), 599 (37%) were women, 766 (48%) had paroxysmal AF, 604 (38%) had persistent AF (< 12 months), and 236 (15%) had long‐standing AF (≥ 12 months). The median Hb level was 13.7 g/dL (IQR 12.4–14.8). Patients with Hb < 12.4 g/dL were classified as the Q1 group, and those with Hb ≥ 12.4 g/dL as the Q2–4 group. Baseline and procedural characteristics are summarized in Table 1. Compared with the Q2–4 group, patients in the Q1 group were older, more often female, and had lower body mass index. They also had higher CHA_2_DS_2_‐VASc scores, more frequent chronic kidney disease, and higher BNP levels. In terms of procedural variables, cavo‐tricuspid isthmus ablation was less frequently performed in Q1, while other ablation strategies, procedure time, and total radiofrequency energy were comparable between groups.

**TABLE 1:** Baseline and procedural characteristics in the RFA cohort.

Variables	Q1 group (Hb < 12.4 g/dL), *n* = 384	Q2‐4 (Hb ≥ 12.4 g/dL), *N* = 1222	*p*
**Baseline characteristics**			
Age	76 (71–80)	70 (63–76)	< 0.001
Female	245 (64)	354 (29)	< 0.001
Body mass index	22.7 (20.3–25.3)	24.2 (22.0–26.8)	< 0.001
AF types			
Paroxysmal	223 (58)	543 (44)	< 0.001
Persistent (< 12 months)	120 (31)	484 (40)	
Long‐standing (≥ 12 months)	41 (11)	195 (16)	
Hypertension	247 (64)	723 (59)	0.073
Diabetes mellitus	70 (18)	204 (17)	0.485
CHA2DS2‐VASc score	4 (3–5)	3 (1–4)	< 0.001
Chronic kidney disease	144 (38)	238 (19)	< 0.001
Left ventricular ejection fraction	67 (60–72)	66 (61–71)	0.526
Left atrial diameter, mm	44 (41–49)	45 (40–49)	0.571
Hemoglobin, g/dL	11.4 (10.7–12.0)	14.3 (13.4–15.1)	< 0.001
BNP, pg/mL	162.8 (72.0–289.5)	98.5 (44.0–188.0)	< 0.001
β blocker	196 (51)	530 (43)	0.010
ACEI or ARB	162 (42)	459 (38)	0.105
**Procedural characteristics**			
Pulmonary vein isolation	384 (100)	1222 (100)	> 0.999
Cavo‐tricuspid isthmus	163 (43)	633 (52)	< 0.001
Superior vena cava	65 (17)	224 (18)	0.594
Left atrial linear ablation	65 (17)	186 (15)	0.421
Non‐pulmonary vein trigger	17 (4.4)	78 (6.4)	0.174
Total procedure time, min	159 (127–200)	161 (128–205)	0.330
Total radiofrequency energy, 1000 J	50.5 (40.1–65.1)	51.9 (39.4–64.9)	0.988

*Note:* Data are given as the *n* (%) or median (interquartile range).

Abbreviations: ACEI, angiotensin converting enzyme inhibitor; AF, atrial fibrillation; ARB, angiotensin II receptor blocker; BNP, brain natriuretic peptide.

**FIGURE 1:**
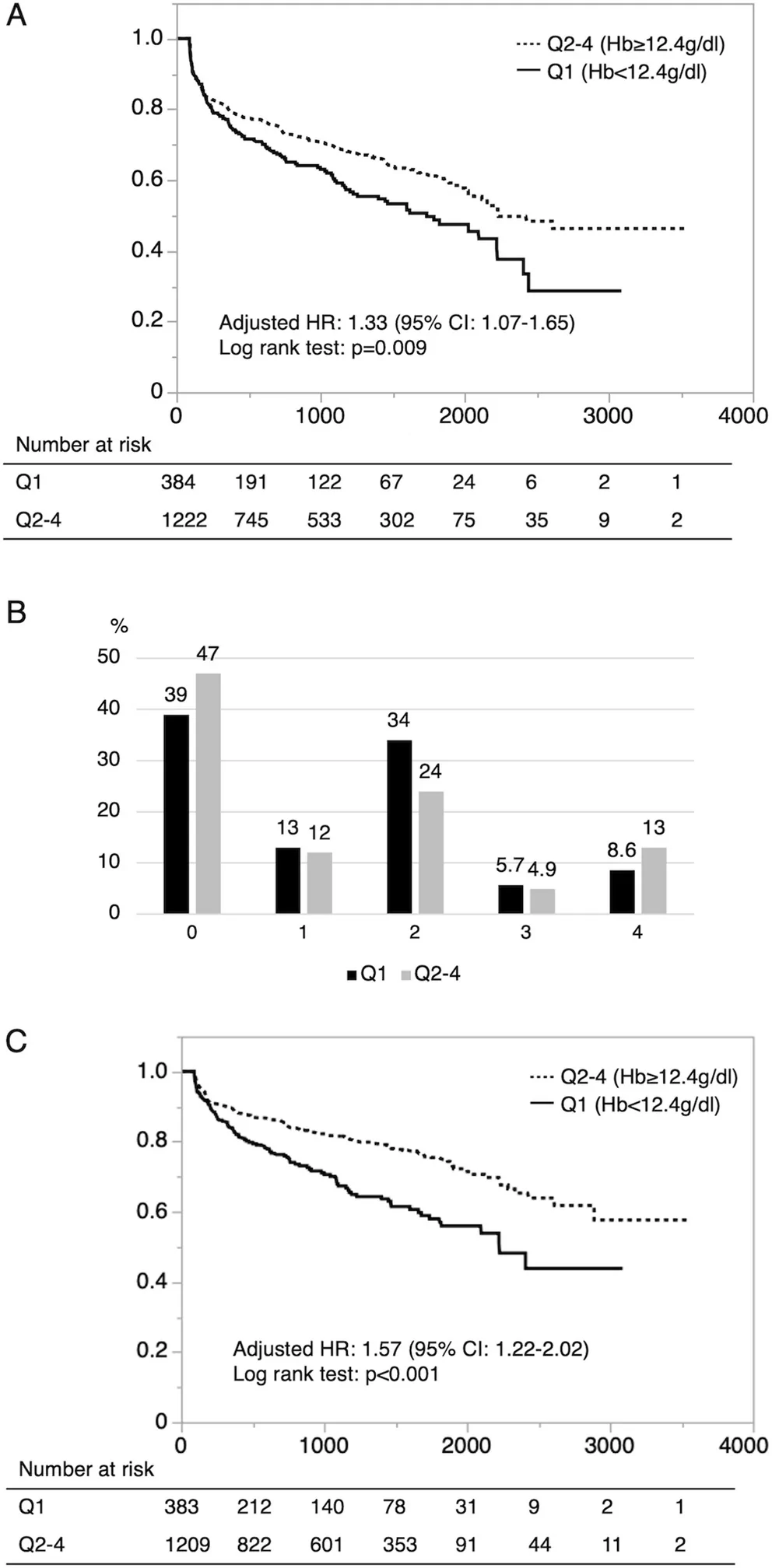
Impact of baseline hemoglobin levels on arrhythmia recurrence after radiofrequency ablation. (A) Kaplan–Meier curves for atrial fibrillation/atrial tachycardia recurrence after the index RFA procedure according to baseline Hb levels. Patients in the lowest quartile (Q1: Hb < 12.4 g/dL) had a significantly higher incidence of recurrence compared with those in the higher quartiles (Q2–4: Hb ≥ 12.4 g/dL) (adjusted HR 1.33, 95% CI 1.07–1.65; log‐rank *p* = 0.009). (B) Distribution of the number of reconnected PVs at the time of repeat ablation after RFA. No significant difference was observed between the Q1 and Q2–4 groups (*p* = 0.383). (C) Kaplan–Meier curves for recurrence after multiple ablation sessions landmarked at the completion of the final procedure. Patients in the Q1 group remained at higher risk of recurrence compared with those in the Q2–4 group (adjusted HR 1.57, 95% CI 1.22–2.02; log‐rank *p* < 0.001). HRs were adjusted for age, sex, AF type, chronic kidney disease, and left atrial diameter. AF, atrial fibrillation; Hb, hemoglobin; PV, pulmonary vein; RFA, radiofrequency ablation.

Patients in the Q1 group (Hb < 12.4 g/dL) had a significantly higher incidence of arrhythmia recurrence after the index procedure compared with those in the Q2–4 group (Hb ≥ 12.4 g/dL) (HR 1.33, 95% CI 1.07–1.65; log‐rank *p* = 0.009, Figure 1A, Table 2). Arrhythmia recurrence after the index procedure occurred in 148 patients in the Q1 group and 392 patients in the Q2–4 group. Restricted cubic spline analysis demonstrated a consistent inverse association between Hb levels and arrhythmia recurrence risk. No evidence of a nonlinear relationship was observed (Figure S1A, *p* for nonlinearity = 0.151), supporting the validity of the cut‐off–based analysis. A similar trend was also observed when hematocrit was analyzed as a continuous variable (Figure S1B, *p* for nonlinearity = 0.095). Redo procedures were subsequently performed in 70 and 245 patients, respectively. The distribution of the number of reconnected PVs at the redo session was similar between the two groups (*p* = 0.383, Figure 1B). In the analysis of patients who underwent multiple sessions and completed a 90‐day blanking period after the final procedure, the Q1 group (Hb < 12.4 g/dL) still showed a higher risk of atrial arrhythmia recurrence compared with the Q2–4 group (HR 1.57, 95% CI 1.22–2.02; log‐rank *p* < 0.001, Figure 1C, Table 2).

**TABLE 2:** Multivariable Cox proportional hazards analysis for arrhythmia recurrence in the RFA cohort.

Variables	HR (95% CI)	*p*
**After single procedure**		
Low Hb level in Q1 group (Hb < 12.4 g/dL)	1.33 (1.07–1.65)	0.012
Age	0.889 (0.483–1.633)	0.704
Female	1.33 (1.10–1.61)	0.003
AF types		
Paroxysmal	Reference	
Persistent (< 12 months)	1.65 (1.35–2.03)	< 0.001
Long‐standing (≥ 12 months)	2.88 (2.27–3.65)	< 0.001
Chronic kidney disease	1.16 (0.94–1.44)	0.172
BNP	1.0005 (1.0001–1.0009)	0.026
Left atrial diameter	1.002 (0.999–1.006)	0.164
**After multiple procedure**		
Low Hb level in Q1 group (Hb < 12.4 g/dL)	1.57 (1.22–2.02)	< 0.001
Age	1.015 (1.003–1.027)	0.016
Female	1.42 (1.12–1.80)	0.004
AF types		
Paroxysmal	Reference	
Persistent (< 12 months)	1.74 (1.34–2.26)	< 0.001
Long‐standing (≥ 12 months)	3.53 (2.64–4.71)	< 0.001
Chronic kidney disease	1.19 (0.92–1.53)	0.193
BNP	1.0006 (1.0000–1.0010)	0.027
Left atrial diameter	1.003 (0.998–1.006)	0.096

Abbreviation: BNP, brain natriuretic peptide.

### 3.2 CBA Cohort

A total of 596 patients were included in the CBA cohort. The median age was 71 years (IQR 61–77), and 204 (34%) were women. A total of 435 patients (73%) had paroxysmal AF, 136 (23%) had persistent AF (< 12 months), and 25 (4%) had long‐standing AF (≥ 12 months). The median Hb level was 13.7 g/dL (IQR 12.6–14.7). Compared with the RFA cohort, patients undergoing CBA were more likely to have paroxysmal AF and exhibited smaller left atrial diameter, lower CHA_2_DS_2_‐VASc scores, and lower BNP levels, all with *p* < 0.001 (Table S1). Hb distribution did not differ significantly between the RFA and CBA cohorts. For consistency with the RFA analysis, patients in the CBA cohort were categorized according to the RFA‐derived quartile cut‐off of 12.4 g/dL: those with Hb < 12.4 g/dL were classified as the Low Hb group (*n* = 132, 22%), and those with Hb ≥ 12.4 g/dL as the non‐low Hb group (*n* = 464, 78%). Baseline and procedural characteristics are summarized in Table 3. Compared with the non‐low Hb group, patients in the Low Hb group were older, more often female, and had lower body mass index. They also had higher CHA_2_DS_2_‐VASc scores, more frequent chronic kidney disease, and higher BNP levels. In terms of procedural variables, procedure time was longer in the Low Hb group, while additional ablation strategies and the acute efficacy of PVI were comparable between groups.

**TABLE 3:** Baseline and procedural characteristics in the CBA cohort.

Variables	Low Hb group (Hb < 12.4 g/dL), *n* = 132	Non‐low Hb group (Hb ≥ 12.4 g/dL), *N* = 464	*p*
**Baseline characteristics**			
Age	76 (70–81)	70 (59–76)	< 0.001
Female	85 (64)	119 (26)	< 0.001
Body mass index	22.5 (20.5–24.7)	24.0 (21.8–26.6)	< 0.001
AF types			
Paroxysmal	111 (84)	324 (70)	0.003
Persistent (< 12 months)	20 (15)	116 (25)	
Long‐standing (≥ 12 months)	1 (0.8)	24 (5.2)	
Hypertension	74 (56)	252 (54)	0.658
Diabetes mellitus	18 (14)	57 (12)	0.682
CHA2DS2‐VASc score	3 (2–4)	2 (1–3)	< 0.001
Chronic kidney disease	32 (24)	60 (13)	0.003
Left ventricular ejection fraction	69 (63–73)	68 (64–72)	0.723
Left atrial diameter, mm	42 (38–47)	42 (38–45)	0.386
Hemoglobin, g/dL	11.7 (10.9–12.1)	14.1 (13.3–14.9)	< 0.001
BNP, pg/mL	89.4 (33.7–203.7)	47.5 (20.5–98.8)	< 0.001
β blocker	59 (45)	155 (33)	0.018
ACEI or ARB	49 (37)	166 (36)	0.837
**Procedural characteristics**			
Pulmonary vein isolation	132 (100)	464 (100)	> 0.999
Cavo‐tricuspid isthmus	3 (2.3)	21 (4.5)	0.321
Total procedure time, min	92 (75–123)	82 (68–105)	0.006

*Note:* Data are given as the *n* (%) or median (interquartile range).

Abbreviations: ACEI, angiotensin converting enzyme inhibitor; AF, atrial fibrillation; ARB, angiotensin II receptor blocker; BNP, brain natriuretic peptide.

**FIGURE 2:**
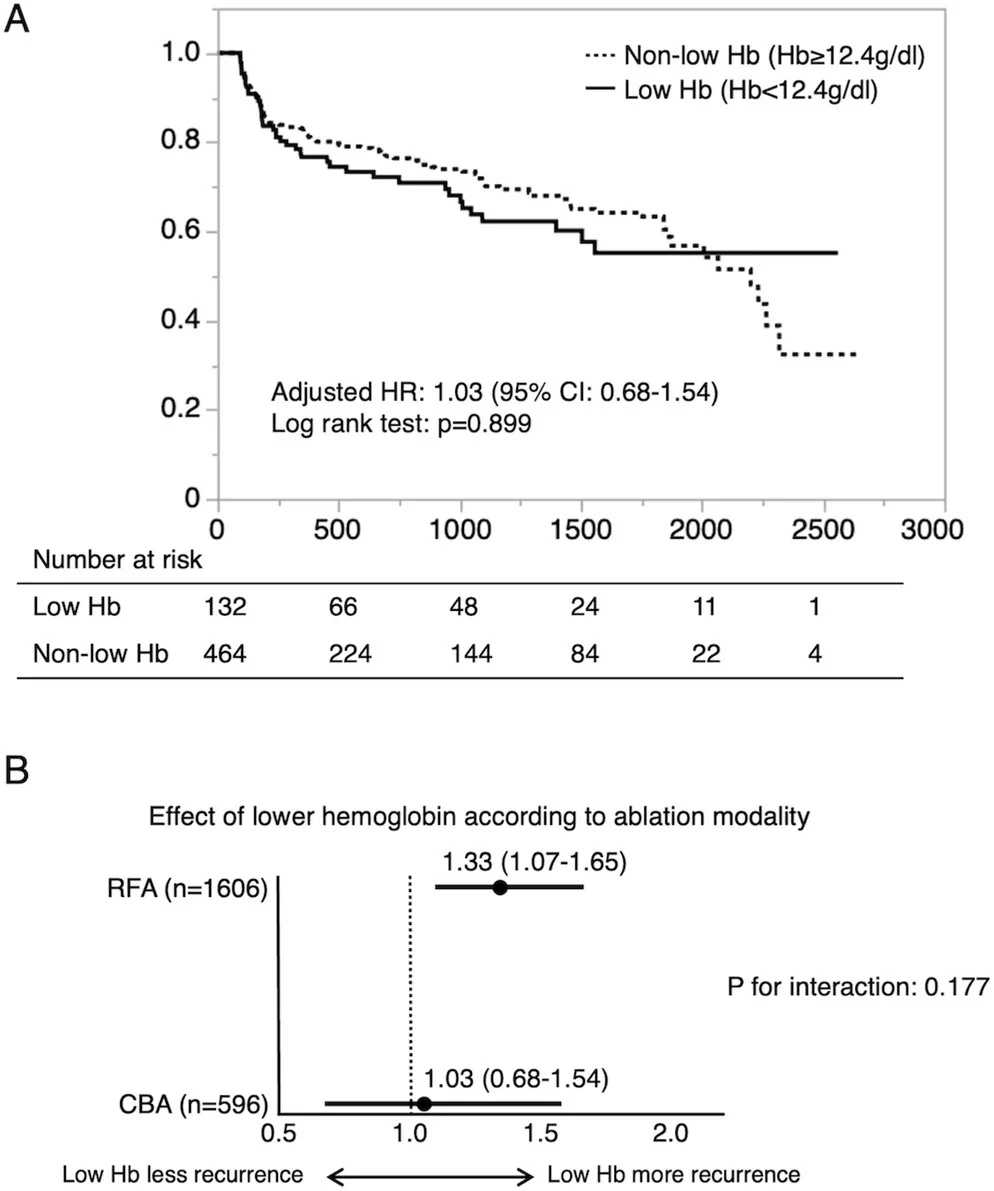
Association between baseline hemoglobin and arrhythmia recurrence after cryoballoon ablation, and effect of lower hemoglobin according to ablation modality. (A) Kaplan–Meier curves for recurrence of atrial fibrillation/atrial tachycardia beyond the 90‐day blanking period in the CBA cohort, comparing the lower‐Hb group (Hb < 12.4 g/dL) and the non‐low Hb group (Hb ≥ 12.4 g/dL). The cut‐off (12.4 g/dL) was derived from the RFA cohort. (B) Forest plot showing adjusted hazard ratios for recurrence (lower‐Hb vs. non‐low Hb) stratified by ablation modality. In the RFA cohort, lower‐Hb was significantly associated with higher recurrence, whereas the association was not significant in the CBA cohort. The modality × hemoglobin interaction was not statistically significant (*p* for interaction = 0.177). HRs were adjusted for age, sex, AF type, chronic kidney disease, BNP and left atrial diameter. CBA, cryoballoon ablation; other abbreviations are shown in Figure 1.

During follow‐up, atrial arrhythmia recurrence occurred in 43 patients (33%) in the Low Hb group and 125 patients (27%) in the non‐low Hb group. Kaplan–Meier analysis showed no significant difference in recurrence between the two groups (log‐rank *p* = 0.407, Figure 2A). In multivariable Cox regression adjusted for age, sex, AF type, chronic kidney disease, BNP, and left atrial diameter, the association between Low Hb and recurrence was not statistically significant (adjusted HR 1.03, 95% CI 0.68–1.54, *p* = 0.899, Table 4). Restricted cubic spline analysis in the CBA cohort did not demonstrate a clear association between Hb level and arrhythmia recurrence (Figure S2).

**TABLE 4:** Multivariable Cox proportional hazards analysis for arrhythmia recurrence in the CBA cohort.

Variables	HR (95% CI)	*p*
**After single procedure**		
Low Hb level (Hb < 12.4 g/dL)	1.03 (0.68–1.54)	0.899
Age	0.997 (0.983–1.012)	0.713
Female	1.46 (1.02–2.09)	0.037
AF types		
Paroxysmal	Reference	
Persistent (< 12 months)	2.49 (1.70–3.65)	< 0.001
Long‐standing (≥ 12 months)	4.86 (2.53–9.32)	< 0.001
Chronic kidney disease	1.12 (0.69–1.79)	0.652
BNP	1.0004 (1.0001–1.0007)	0.002
Left atrial diameter	1.005 (0.994–1.011)	0.217

Abbreviation: BNP, brain natriuretic peptide.

### 3.3 Total Population

In the pooled analysis including both RFA and CBA cohorts, multivariable Cox regression adjusted for age, sex, AF type, chronic kidney disease, BNP, left atrial diameter, and ablation modality (RFA or CBA) showed that low Hb was independently associated with arrhythmia recurrence (adjusted HR 1.24, 95% CI 1.03–1.50; *p* = 0.023, Table 5). In patients with non‐paroxysmal AF, low Hb remained independently associated with arrhythmia recurrence after adjustment for age, sex, chronic kidney disease, BNP, left atrial diameter, and ablation modality (adjusted HR 1.43, 95% CI 1.11–1.85; *p* = 0.006, Table 5), whereas low Hb was not associated with arrhythmia recurrence (adjusted HR 1.06, 95% CI 0.80–1.39; *p* = 0.694, Table 5) in patients with paroxysmal AF. Finally, in the pooled analysis including both RFA and CBA cohorts, the interaction between ablation modality and Hb group was not significant (*p* for interaction = 0.177, Figure 2B). Sensitivity analysis using the WHO definition of anemia yielded findings consistent with the previously described analyses (Table S2).

**TABLE 5:** Multivariable Cox proportional hazards analysis for arrhythmia recurrence in the total population.

Population	Adjusted HR for low Hb (95% CI)	*p*
Total population	1.24 (1.03–1.50)	0.023
Non‐paroxysmal AF	1.43 (1.11–1.85)	0.006
Paroxysmal AF	1.06 (0.80–1.39)	0.694

*Note:* The total population model was adjusted for age, sex, AF type, chronic kidney disease, BNP, left atrial diameter, and ablation modality. Analyses stratified by AF type were adjusted for age, sex, chronic kidney disease, BNP, left atrial diameter, and ablation modality.

## 4 Discussion

### 4.1 Main Findings

In this large single‐center cohort, we found that (1) lower Hb levels were significantly associated with arrhythmia recurrence after RFA and in the total population, but not after CBA; (2) after multiple sessions in which PV reconnection was minimized, lower Hb (Q1 group) remained an independent predictor of recurrence in the RFA cohort, while the number of PV reconnections in the redo session did not differ between Hb Q1 and Q2–4 groups; and (3) the interaction between ablation modality and lower Hb (Q1 group) was not statistically significant for arrhythmia recurrence.

### 4.2 Low Hb Levels: Implications for Atrial Substrate Progression

In the present study, lower Hb levels were independently associated with arrhythmia recurrence after AF ablation, even after adjustment for established clinical and echocardiographic risk factors, including age, sex, AF type, chronic kidney disease, BNP, and left atrial diameter. Importantly, this association was observed not only in the RFA cohort but also in the pooled analysis across ablation modalities, supporting the concept that Hb reflects a risk profile not fully captured by conventional markers of atrial disease severity. A prior nationwide study from Korea also reported that anemia was an independent predictor of AF recurrence after ablation [13]. Unlike our study, that report did not specifically address mechanisms related to PV reconnection or ablation modality. Accumulating evidence indicates that anemia and lower Hb levels are associated with adverse cardiovascular remodeling [6, 14], and left atrial enlargement [14, 15], which may contribute to atrial cardiomyopathy [7]. These observations support the concept that lower Hb may reflect progression of an adverse atrial substrate and create a substrate more vulnerable to recurrence, even after durable PVI.

### 4.3 Impact of Low Hemoglobin Levels on Lesion Formation

Lesion formation with RFA depends on resistive heating, and effective energy delivery is influenced by tissue and blood impedance. Reduced Hb lowers blood impedance, which facilitates current shunting into the blood pool and thereby limits effective tissue heating. Experimental studies have shown that lesions created under low‐impedance conditions are smaller and shallower [8, 9]. In contrast, CBA delivers cryothermal energy largely independent of blood conductivity; lesion durability is determined mainly by balloon–tissue contact and cooling efficiency, as reflected by parameters such as time‐to‐isolation, nadir balloon temperature, and thawing duration [10]. Consistent with these mechanistic differences, our single‐session analysis demonstrated that lower Hb was associated with higher recurrence after RFA, whereas no significant association was observed after CBA.

However, in the RFA cohort, arrhythmia recurrence remained more frequent in the lower Hb group (Q1) even after multiple sessions. Moreover, among patients who underwent repeat ablation, the number of reconnected PVs did not differ significantly between Hb Q1 and Q2–4 groups. These results suggest that the contribution of impedance‐related RFA energy loss to arrhythmia recurrence is likely limited. This interpretation should be made with caution, as only a subset of patients with recurrence underwent redo procedures (70 of 148 in the low‐Hb group and 245 of 392 in the non‐low‐Hb group), and PV reconnection may also exist in patients without documented clinical recurrence. The weaker association between lower Hb (Q1) and clinical outcomes in the CBA cohort compared with the RFA cohort may be explained by differences in the underlying atrial substrate. Patients treated with RFA more frequently exhibited features of advanced atrial disease, including a higher prevalence of non‐paroxysmal AF, larger left atrial size, and elevated BNP levels. In this population, in whom PVI alone may be less sufficient to prevent recurrence, lower Hb may function as a surrogate marker of more advanced atrial remodeling rather than as a purely procedural modifier. This interpretation was supported by our subgroup analysis in total population according to AF type, in which low Hb remained independently associated with arrhythmia recurrence only in patients with non‐paroxysmal AF, but not in those with paroxysmal AF.

### 4.4 Clinical Implications

Our findings suggest that Hb concentration serves primarily as a marker of an adverse atrial substrate associated with arrhythmia recurrence after AF ablation, particularly in patients with more advanced atrial disease. The persistence of the association between low Hb and recurrence even after repeat procedures supports the concept that Hb reflects progression of atrial remodeling beyond PVI. From a clinical perspective, this implies that Hb may be relevant for risk stratification in selected patient populations, such as those undergoing RFA with features of advanced atrial remodeling, rather than across all ablation candidates. In this context, Hb may also provide complementary information in patients treated with emerging pulsed field ablation technologies, in whom lesion durability is less dependent on blood conductivity. Given that Hb is routinely measured in clinical practice, it represents a simple and inexpensive marker that could be incorporated into pre‐procedural assessment and long‐term management in appropriately selected patients.

### 4.5 Limitations

First, this was a retrospective, single‐center study, and unmeasured confounders cannot be excluded despite multivariable adjustment. Second, Hb was chosen as the main hematological parameter rather than hematocrit because it reflects both oxygen‐carrying capacity and blood conductivity. Nevertheless, a supplementary spline analysis using hematocrit showed a similar inverse trend, supporting the robustness of our findings. Third, arrhythmia recurrence was assessed by scheduled Holter recordings and intermittent ECGs without continuous monitoring, such as implantable cardiac monitoring. As a result, asymptomatic or brief episodes may have been missed. Fourth, only a subset of patients with recurrence underwent repeat ablation, and PV reconnection can also exist in patients without clinical recurrence; thus, the analysis of PV reconnection may not fully reflect the relationship between Hb and lesion durability. Fifth, because of the retrospective nature of this study, the underlying causes of low Hb were not systematically evaluated. Therefore, we were unable to determine the contribution of individual factors, including iron deficiency, chronic kidney disease, inflammation, malignancy, and bleeding, to the observed association with arrhythmia recurrence. Finally, the observational design precludes any conclusions regarding causality between Hb levels and arrhythmia recurrence.

## 5 Conclusions

In this large single‐center cohort, lower Hb levels were independently associated with a higher risk of arrhythmia recurrence after RFA, but not after CBA. The persistence of this association even after repeat procedures suggests that the impact of low Hb is not primarily explained by impedance‐related procedural factors. In addition, in the pooled analysis, the association between low Hb and arrhythmia recurrence differed according to AF type, suggesting that low Hb may reflect progression of an adverse atrial substrate rather than a procedural effect.

## Funding

The authors have nothing to report.

## Ethics Statement

The study was approved by the local institutional ethics committee and conducted in accordance with the Declaration of Helsinki (reference no. 2025‐70).

## Conflicts of Interest

The authors declare no conflicts of interest.

## Supporting information


**Figure S1:** Restricted cubic spline curves in the RFA cohort for hemoglobin (A) and hematocrit (B) levels in relation to arrhythmia recurrence risk in the Cox proportional hazards model, adjusted for age, sex, AF type, CKD, BNP and LA diameter. The reference was set at the median value (13.7 g/dL for hemoglobin and 40.4% for hematocrit). Both analyses showed a consistent inverse trend without significant evidence of nonlinearity (A: *p* for nonlinearity = 0.151; B: *p* for nonlinearity = 0.095).

**Figure S2:** Restricted cubic spline curve in the CBA cohort for hemoglobin levels in relation to arrhythmia recurrence risk in the Cox proportional hazards model, adjusted for age, sex, AF type, CKD, BNP and LA diameter. The reference was set at the median value (13.6 g/dL).

**Table S1:** Baseline and procedural characteristics of the RFA and CBA cohorts.

**Table S2:** Sensitivity analysis using the WHO definition of anemia (< 13 g/dL in men and < 12 g/dL in women).

## Data Availability

The data that support the findings of this study are available from the corresponding author upon reasonable request.

## References

[1] J. A. Joglar , M. K. Chung , A. L. Armbruster , et al., “2023 ACC/AHA/ACCP/HRS Guideline for the Diagnosis and Management of Atrial Fibrillation: A Report of the American College of Cardiology/American Heart Association Joint Committee on Clinical Practice Guidelines,” Circulation 149, no. 1 (2024): e1–e156. 10.1161/CIR.0000000000001193 PMC11095842 38033089

[2] M. Masuda , K. Inoue , N. Tanaka , et al., “Long‐Term Impact of Additional Ablation After Pulmonary Vein Isolation: Results From EARNEST‐PVI Trial,” Journal of the American Heart Association 12, no. 17 (2023): e029651. 10.1161/JAHA.123.029651 PMC10547359 37642022

[3] Y. Matsunaga‐Lee , K. Inoue , N. Tanaka , et al., “Duration of Atrial Fibrillation Persistence: Implications for Recurrence Risk After Catheter Ablation and Efficacy of Additional Substrate Ablation,” Heart Rhythm 21, no. 6 (2024): 733–740. 10.1016/j.hrthm.2024.01.053 38307310

[4] Y. Matsunaga‐Lee , K. Inoue , N. Tanaka , et al., “Risk Factors of Late Arrhythmia Recurrences Over 12 Months After Catheter Ablation of Atrial Fibrillation: Insight Into Atrial Cardiomyopathy,” Heart Rhythm 22 (2024): 3007–3009. 10.1016/j.hrthm.2024.12.018 39701441

[5] P. A. Heidenreich , B. Bozkurt , D. Aguilar , et al., “2022 AHA/ACC/HFSA Guideline for the Management of Heart Failure: A Report of the American College of Cardiology/American Heart Association Joint Committee on Clinical Practice Guidelines,” Circulation 145, no. 18 (2022): e895–e1032. 10.1161/CIR.0000000000001063 35363499

[6] N. Grote Beverborg , D. J. van Veldhuisen , and P. van der Meer , “Anemia in Heart Failure: Still Relevant?,” JACC: Heart Failure 6, no. 3 (2018): 201–208. 10.1016/j.jchf.2017.08.023 29128254

[7] Y. N. V. Reddy , M. Obokata , F. H. Verbrugge , G. Lin , and B. A. Borlaug , “Atrial Dysfunction in Patients With Heart Failure With Preserved Ejection Fraction and Atrial Fibrillation,” Journal of the American College of Cardiology 76, no. 9 (2020): 1051–1064. 10.1016/j.jacc.2020.07.009 PMC7455760 32854840

[8] H. Nakagawa , F. H. M. Wittkampf , and W. M. Jackman , “Can we Produce Deeper Radiofrequency Lesions?,” JACC. Clinical Electrophysiology 3, no. 10 (2017): 1111–1113. 10.1016/j.jacep.2017.06.002 29759493

[9] N. Ariyarathna , S. Kumar , S. P. Thomas , W. G. Stevenson , and G. F. Michaud , “Role of Contact Force Sensing in Catheter Ablation of Cardiac Arrhythmias: Evolution or History Repeating Itself?,” JACC. Clinical Electrophysiology 4, no. 6 (2018): 707–723. 10.1016/j.jacep.2018.03.014 29929663

[10] S. Miyazaki , T. Kajiyama , T. Watanabe , et al., “Predictors of Durable Pulmonary Vein Isolation After Second‐Generation Cryoballoon Ablation With a Single Short Freeze Strategy ‐ Different Criteria for the Best Freeze of the 4 Individual PVs,” International Journal of Cardiology 301 (2020): 96–102. 10.1016/j.ijcard.2019.11.089 31759685

[11] Y. Matsunaga‐Lee , Y. Egami , M. Ohsuga , et al., “Novel Tailored Very‐High‐Power Short‐Duration Radiofrequency Ablation Around the Esophagus Guided by Left Atrial Voltage,” Journal of Cardiology 86 (2025): 249–255. 10.1016/j.jjcc.2025.03.005 40086665

[12] K. Ukita , Y. Egami , S. Kawanami , et al., “Impact of Very Early Recurrence of Atrial Fibrillation After Cryoballoon Ablation,” Pacing and Clinical Electrophysiology 45, no. 11 (2022): 1323–1329. 10.1111/pace.14583 35959745

[13] M. Kim , M. Hong , J. Y. Kim , et al., “Clinical Relationship Between Anemia and Atrial Fibrillation Recurrence After Catheter Ablation Without Genetic Background,” International Journal of Cardiology Heart & Vasculature 27 (2020): 100507. 10.1016/j.ijcha.2020.100507 PMC7125353 32258364

[14] Y. Ying , J. Ye , Z. Yuan , and D. Cai , “Association of Anaemia on Heart Failure and Left Ventricular Function: A Bidirectional Mendelian Randomization Study,” ESC Heart Failure 11, no. 1 (2024): 299–305. 10.1002/ehf2.14579 PMC10804204 37984882

[15] T. Katayama , N. Fujiwara , and Y. Tsuruya , “Factors Contributing to Left Atrial Enlargement in Adults With Normal Left Ventricular Systolic Function,” Journal of Cardiology 55, no. 2 (2010): 196–204. 10.1016/j.jjcc.2009.10.008 20206072

