# Supplementary material for: Hemoglobin Levels as a Marker of Arrhythmia Recurrence After Atrial Fibrillation Ablation
Source: J Arrhythm. 2026 Sep 21;42(5):e70473. doi: 10.1002/joa3.70473 (PMC13594780; doi:10.1002/joa3.70473)
Supplement: Supplementary file 1 [file JOA3-42-e70473-s001.docx]

**Supplementary Figure 2**

**Supplementary Figure 2.** Restricted cubic spline curve in the CBA cohort for hemoglobin levels in relation to arrhythmia recurrence risk in the Cox proportional hazards model, adjusted for age, sex, AF type, CKD, BNP and LA diameter. The reference was set at the median value (13.6 g/dL).
