# Supplementary material for: Hemoglobin Levels as a Marker of Arrhythmia Recurrence After Atrial Fibrillation Ablation
Source: J Arrhythm. 2026 Sep 21;42(5):e70473. doi: 10.1002/joa3.70473 (PMC13594780; doi:10.1002/joa3.70473)
Supplement: Supplementary file 2 [file JOA3-42-e70473-s002.docx]

| **Supplementary Table1: Baseline and procedural characteristics of the RFA and CBA cohorts** | | | |  |
| --- | --- | --- | --- | --- |
| Variables | RFA cohort  n=1606 | CBA cohort  N=596 | P value |  |
| Baseline characteristics |  |  |  |  |
| Age | 72 (65-77) | 71 (61-78) | 0.110 |  |
| Female | 599 (37) | 204 (34) | 0.195 |  |
| Body mass index | 23.8 (21.6-26.5) | 23.7 (21.5-26.3) | 0.237 |  |
| AF types |  |  | <0.001 |  |
| Paroxysmal | 766 (48) | 435 (73) |  |  |
| Persistent (<12 months) | 604 (38) | 136 (23) |  |  |
| Long-standing (≥12 months) | 236 (15) | 25 (4.2) |  |  |
| Hypertension | 970 (60) | 326 (55) | 0.019 |  |
| Diabetes mellitus | 274 (17) | 75 (13) | 0.010 |  |
| CHA2DS2-VASc score | 3 (2-4) | 2 (1-4) | <0.001 |  |
| Chronic kidney disease | 382 (24) | 92 (15) | <0.001 |  |
| Left ventricular ejection fraction | 66 (61-71) | 68 (64-72) | <0.001 |  |
| Left atrial diameter, mm | 45 (40-49) | 42 (38-46) | <0.001 |  |
| Hemoglobin, g/dl | 13.7 (12.4-14.8) | 13.6 (12.5-14.6) | 0.635 |  |
| BNP, pg/mL | 111.1 (48.5-213.0) | 52.1 (23.5-115.8) | <0.001 |  |
| β blocker | 726 (45) | 214 (36) | <0.001 |  |
| ACEI or ARB | 621 (39) | 215 (36) | 0.277 |  |

Data are given as the n (%) or median (Interquartile range). ACEI, Angiotensin converting enzyme inhibitor; AF: atrial fibrillation; ARB, Angiotensin Ⅱ receptor blocker; BNP, brain natriuretic peptide; CBA, cryoballoon ablation; RFA, radiofrequency ablation.
