# Supplementary material for: Hemoglobin Levels as a Marker of Arrhythmia Recurrence After Atrial Fibrillation Ablation
Source: J Arrhythm. 2026 Sep 21;42(5):e70473. doi: 10.1002/joa3.70473 (PMC13594780; doi:10.1002/joa3.70473)
Supplement: Supplementary file 3 [file JOA3-42-e70473-s003.docx]

**Supplementary Figure 1**

**Supplementary Figure 1.** Restricted cubic spline curves in the RFA cohort for hemoglobin (A) and hematocrit (B) levels in relation to arrhythmia recurrence risk in the Cox proportional hazards model, adjusted for age, sex, AF type, CKD, BNP and LA diameter. The reference was set at the median value (13.7 g/dL for hemoglobin and 40.4% for hematocrit). Both analyses showed a consistent inverse trend without significant evidence of non-linearity (A: p for non-linearity = 0.151; B: p for non-linearity = 0.095).
