# Supplementary material for: Hemoglobin Levels as a Marker of Arrhythmia Recurrence After Atrial Fibrillation Ablation
Source: J Arrhythm. 2026 Sep 21;42(5):e70473. doi: 10.1002/joa3.70473 (PMC13594780; doi:10.1002/joa3.70473)
Supplement: Supplementary file 4 [file JOA3-42-e70473-s004.docx]

| **Supplementary Table2:** Sensitivity analysis using the WHO definition of anemia (<13 g/dL in men and <12 g/dL in women). | | | |  |
| --- | --- | --- | --- | --- |
| Population | Adjusted HR for WHO anemia (95% CI) | p value |  |  |
| Total population | 1.34 (1.12-1.60) | 0.002 |  |  |
| RFA single procedure | 1.34 (1.09-1.65) | 0.005 |  |  |
| RFA multiple procedure | 1.52 (1.19-1.93) | 0.001 |  |  |
| CBA single procedure | 1.30 (0.88-1.92) | 0.190 |  |  |
| Non-paroxysmal AF | 1.53 (1.20-1.95) | 0.001 |  |  |
| Paroxysmal AF | 1.11 (0.85-1.46) | 0.452 |  |  |

The total population model was adjusted for age, sex, AF type, chronic kidney disease, BNP, left atrial diameter, and ablation modality. Analyses stratified by energy source were adjusted for age, sex, AF type, chronic kidney disease, BNP, and left atrial diameter. Analyses stratified by AF type were adjusted for age, sex, chronic kidney disease, BNP, left atrial diameter, and ablation modality.
